# Timing of Imaging after D-Luciferin Injection Affects the Longitudinal Assessment of Tumor Growth Using In Vivo Bioluminescence Imaging

**DOI:** 10.1155/2010/471408

**Published:** 2010-07-05

**Authors:** Yusuke Inoue, Shigeru Kiryu, Makoto Watanabe, Arinobu Tojo, Kuni Ohtomo

**Affiliations:** ^1^Department of Radiology, Institute of Medical Science, University of Tokyo, 4-6-1 Shirokanedai, Minato-ku, Tokyo 108-8639, Japan; ^2^Division of Molecular Therapy, Advanced Clinical Research Center, Institute of Medical Science, University of Tokyo, Tokyo 108-8639, Japan; ^3^Department of Radiology, Graduate School of Medicine, University of Tokyo, Tokyo 113-0033, Japan

## Abstract

The peak signal or the signal at a predetermined, fixed time point after D-luciferin injection may be used for the quantitative analysis of in vivo bioluminescence imaging. We repeatedly performed sequential bioluminescence imaging after subcutaneous injection of D-luciferin in mice bearing subcutaneous tumors. The peak time in each measurement became shorter early after cell inoculation, presumably due to gradual establishment of intratumoral vasculature, and reached a plateau of about 10 min on day 10. Although the correlation between the signal at a fixed time point and the peak signal was high, the signal at 5 or 10 min normalized for the peak signal was lower for earlier days, which caused overestimation of tumor growth. The time course of the signals after D-luciferin injection may vary with time after cell inoculation, and this variation should be considered when determining the imaging protocol for quantitative bioluminescence tumor monitoring.

## 1. Introduction

In vivo bioluminescence imaging (BLI) allows the evaluation of the magnitude and distribution of the expression of the luciferase gene in intact small animals and is widely used for monitoring tumor model animals [1, 2]. In vivo BLI is noninvasive and permits the longitudinal assessment of disease progression and therapeutic effects in a given animal, which improves the reliability and efficiency of experiments. In addition to its convenience and excellent sensitivity, its capability for quantitative assessment is a crucial advantage of in vivo BLI for tumor monitoring [3–6].

In bioluminescence tumor monitoring, mice are usually inoculated with tumor cells that stably express firefly luciferase and then are given intravenous, intraperitoneal, or subcutaneous injections of d-luciferin, the substrate for firefly luciferase [7, 8]. d-Luciferin, administered intraperitoneally or subcutaneously, is absorbed into the blood, is delivered by the blood flow, enters the tumor cells, and is oxidized by luciferase, resulting in light emission. The bioluminescence signal increases gradually, reaches a peak 10–20 min after injection, and then decreases gradually. The peak signal, the signal intensity at the peak, is used as a quantitative indicator of the luciferase activity and, consequently, of the tumor burden. However, sequential imaging after d-luciferin injection is required to determine the peak signal. To improve the throughput of the measurements, many researchers perform image acquisition at a single, predetermined time point after d-luciferin injection and use the signal on the image for quantitative analysis. The peak time depends on various factors, including the location of the tumor, injection route, and injection dose [7–9], and the timing of imaging is determined for each experimental protocol, based on the peak time estimated in preliminary experiments. It has been reported that the signal at a fixed time point and the peak signal similarly represent the tumor burden [8, 10, 11], justifying the single-point imaging strategy. However, prolongation of the peak time has been shown after the administration of an antivascular agent [12]. Although the peak time has been reported to be independent of tumor size [10], differences in the peak time have been observed on different days after cell inoculation in other studies [13, 14]. Tumor growth and therapeutic intervention may affect the time course of the bioluminescence signal after d-luciferin injection and, consequently, the relationship between the peak signal and the signal at a fixed time point in a given tumor.

In this paper, we performed sequential imaging after d-luciferin injection in mice bearing subcutaneous tumors and observed tumor growth longitudinally. We selected subcutaneous injection as the administration route for d-luciferin because intraperitoneal injection can rarely cause intrabowel injection, resulting in erroneously weak signal, while subcutaneous injection is free from the risk of injection failure [8]. The time course of the bioluminescence signal intensity after d-luciferin injection was evaluated, and the relationship between the peak signal and the signal at a fixed time point and its change with days after cell inoculation were determined. The aim of this paper was to investigate the effect of imaging timing on the quantitative results of bioluminescence tumor monitoring.

## 2. Materials and Methods

### 2.1. Cell Lines

The human colon cancer cell line HCT116 was transfected with the firefly luciferase gene using the retroviral method described previously [8, 15]. The cells were named HCT116-Luc cells and were maintained in McCoy**'**s 5A medium (Invitrogen, Grand Island, NY) supplemented with 10% fetal bovine serum (JRH Biosciences, Lenexa, KS) and 1% penicillin/streptomycin (Invitrogen). Cell cultures were incubated at 37°C under 5% CO_2_. Firefly luciferase was stably expressed under the control of the long-terminal repeat of Moloney murine leukemia virus in these cells.

### 2.2. Animals

Six 8-week-old female BALB/c nu/nu mice were inoculated subcutaneously in the dorsal flank with 5 × 10^5^ HCT116-Luc cells mixed with Matrigel (BD Biosciences, San Jose, CA). The mice were obtained from SLC Japan (Hamamatsu, Japan) and handled in accordance with the guidelines of the Institute of Medical Science, University of Tokyo. The experiments were approved by the committee for animal research at the institution.

### 2.3. In Vivo BLI

In vivo BLI was performed using a cooled charge-coupled device camera system (IVIS Imaging System 100; Xenogen/Caliper Life Sciences, Alameda, CA) 3, 5, 7, 10, 12, 14, 19, 21, 24, and 28 days after the inoculation of HCT116-Luc cells. Mice were injected with 75 mg/kg d-luciferin (Beetle Luciferin Potassium Salt, Promega, Madison, WI) in 100 *μ*L of phosphate-buffered saline subcutaneously near the scapula and were placed in the light-tight chamber of the imaging system under isoflurane anesthesia. Beginning 5 min after injection, dorsal luminescent images with an exposure time of 1 s were acquired sequentially at a rate of one image per min until 20 min after d-luciferin injection. Data acquisition was continued until 40 min postinjection on days 3 or 5 and until 25 min on day 7, because of the prolonged time course of light emission. Binning was 4 and the field of view was 15 cm.

### 2.4. Data Analysis

An elliptical region of interest (ROI) was placed over the tumor, and the total signal in the ROI (photons/s) was quantified using Living Image software (version 2.50; Xenogen/Caliper Life Sciences). The same ROI was applied to all images acquired sequentially in a single-imaging session for a given mouse. The total signal intensity was plotted against the time after d-luciferin injection to generate a time-intensity curve. The peak time was determined from the time-intensity curve, and the changes in the peak time with days after tumor cell inoculation were evaluated. The peak signal was defined from the curve as an indicator of tumor burden. In addition to the peak signal, the signals at fixed time points (5, 10, 15, and 20 min) after d-luciferin injection were determined as alternatives to the peak signal. The signal in a given time-intensity curve was normalized for the peak signal in the curve to represent the pattern of temporal changes after d-luciferin injection. The normalized signal at a fixed time point was assessed in relation to the number of days after cell inoculation to evaluate the usefulness of the signal at a fixed time point as an alternative to the peak signal. The signal was divided by the signal obtained on day 3 in the respective animal to calculate the growth ratio as an indicator of tumor growth. The growth ratio was plotted against days after cell inoculation and was compared among values obtained at different time points after d-luciferin injection. In addition, the signal at a fixed time point was compared to the peak signal in the respective time-intensity curve, using all curves obtained from the six mice on various days (*n* = 60). Linear regression analysis was performed by the least-squares method after logarithmic transformation.

## 3. Results

**Figure 1:**
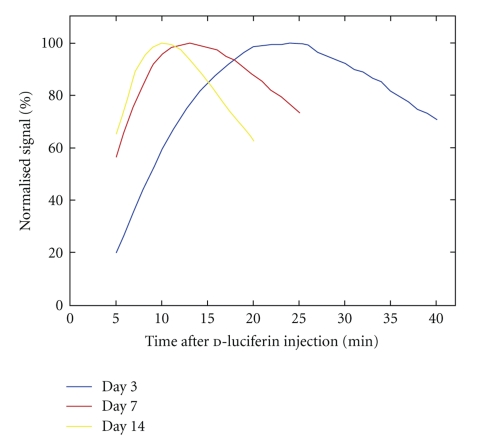
Representative time-intensity curves in a mouse obtained 3, 7, and 14 days after cell inoculation. The signal normalized for the peak signal of the respective curve was plotted against time after d-luciferin injection.

**Figure 2:**
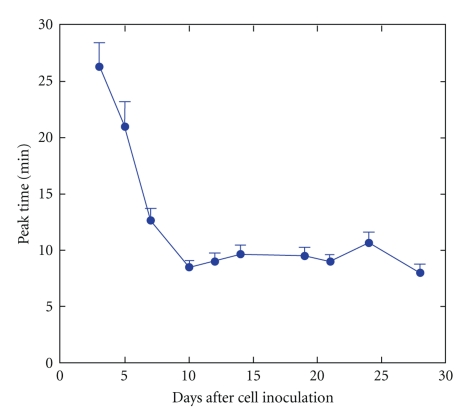
Peak time plotted against days after cell inoculation. The error bars indicate the standard errors.

The subcutaneous tumors were visualized as bright areas on the luminescent images on day 3, and the bioluminescence signals intensified with days after tumor cell inoculation. The tumor diameter increased from about 5 mm on day 7 to about 15 mm on day 28. On day 3, the bioluminescence signal increased slowly after subcutaneous injection of d-luciferin (Figure 1) reached a peak with a mean peak time of 26.3 min (Figure 2), and then decreased. Subsequently, the initial increase became more rapid and the peak time was shortened. On day 10 or later, the mean peak time was almost constant at around 10 min.

**Figure 3:**
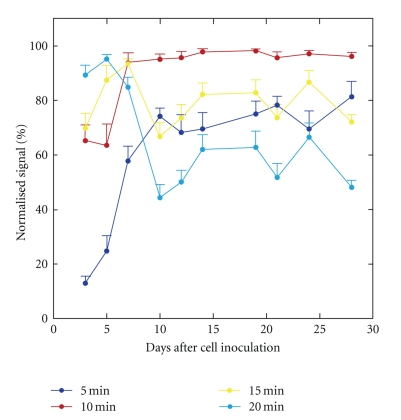
Relationship between days after cell inoculation and the normalized signal. The signal obtained at a fixed time point after d-luciferin injection was normalized for the peak signal. The error bars indicate the standard errors.

On day 3, the signal at 5 min was much lower than the peak signal, consistent with the long peak time (Figure 1). The mean normalized signal at 5 min was 12.9% on day 3 and increased to about 70% on day 10 or later (Figure 3). The signal at 10 min well approximated the peak signal on day 7 or later, leading to a mean normalized signal of more than 95%. However, the mean normalized signal at 10 min was only 65.2% and 63.5% on days 3 and 5, respectively. The signal at 15 min was obviously lower than the peak signal; however, the normalized signal at 15 min did not show a consistent increase or decrease over days. The normalized signal at 20 min was high on days 3–7 and decreased substantially from day 10 on, due to a significant reduction in the signal after the peak.

**Figure 4:**
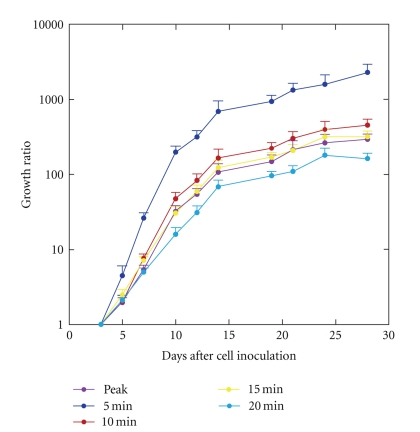
The growth ratio calculated using the signals obtained at various time points after d-luciferin injection. The growth ratios are plotted on a logarithmic scale. The error bars indicate the standard errors.

The peak signal increased over days after cell inoculation, consistent with tumor growth, and the mean growth ratio calculated from the peak signal was 106.9 on day 14 and 294.7 on day 28 (Figure 4). The growth ratio was much larger using the signal at 5 min than when using the other indicators of bioluminescence signal intensity. The mean growth ratio calculated from the signal at 5 min was 692.6 on day 14 and 2302.4 on day 28. The overestimation of the growth ratio was reduced markedly but was still evident using the signal at 10 min (165.5 on day 14, and 455.9 on day 28). The temporal profile of the growth ratio using the signal at 15 min was matched well with that observed using the peak signal. The growth ratio was underestimated using the signal at 20 min.

**Figure 5:**
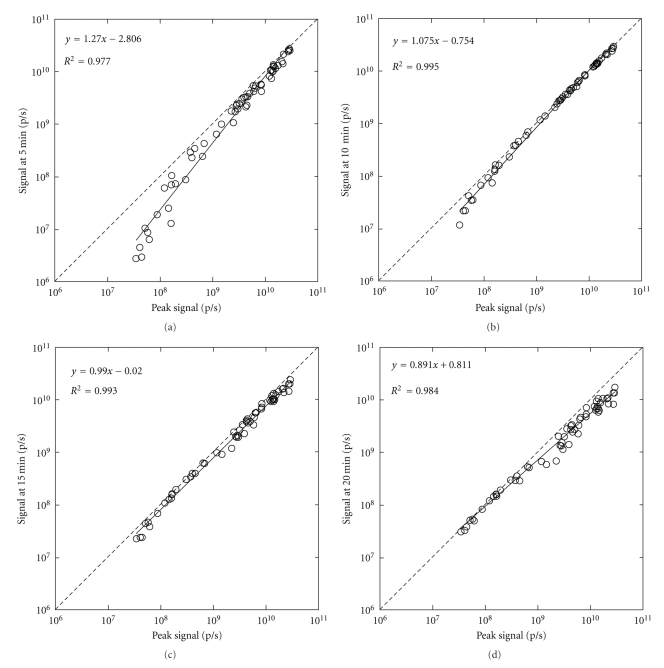
Correlation of the signals at various time points after d-luciferin injection with the peak signal. The signals are presented on a logarithmic scale. The solid and broken lines represent the regression line and the line of identity, respectively. Linear regression was performed after logarithmic transformation.

The signal at a fixed time point after d-luciferin injection was closely correlated with the peak signal, irrespective of the time point used for analysis (Figure 5). The correlation coefficient was higher using the signal at 10 or 15 min than when using the signal at 5 or 20 min. As a result of the underestimation on day 3, weak signals tended to be underestimated using the signal at 5 min, and the discrepancy between the regression line and the line of identity was relatively large.

## 4. Discussion

In this paper, we assessed the time course of the bioluminescence signal after d-luciferin injection in relation to the number of days after tumor cell inoculation and investigated the effect of imaging timing on quantitative tumor monitoring using in vivo BLI. The initial increase in the signal after subcutaneous d-luciferin injection was slow, and the peak time was long early after tumor cell inoculation. The initial increase became more rapid and the peak time was shortened as the number of days after inoculation increased, reaching a plateau on day 10. The time course of the bioluminescence signals during each measurement appears to depend mainly on the rate of absorption of d-luciferin into the blood and the rate of its delivery to the tumor by the blood flow. Since the absorption rate is unlikely to differ with days after cell inoculation, the change in blood supply to the tumor is likely responsible for the changes in the time course of the bioluminescence signals. We speculate that the delivery of d-luciferin to the tumor was slow early after cell inoculation due to insufficient vascularization, which made the elevation of intratumoral d-luciferin concentration timeconsuming. Later, the establishment of the tumor vasculature likely enhanced the delivery of d-luciferin to the tumor, which shortened the peak time. In vivo, BLI is highly sensitive to luciferase-expressing cells and detects them even early after cell inoculation; however, one should note that the time course of the signal after d-luciferin injection may be delayed because the blood supply to the tumor has not been established early after cell inoculation.

In this paper, we regarded the peak signal as the standard and evaluated the usefulness of the signal at a predetermined, fixed time point after d-luciferin injection as an alternative to the peak signal. Ideally, the signal at the fixed time point would be 100% after normalization for the peak signal. If the normalized signal is substantially less than 100% but stable over days after cell inoculation, the signal at a fixed time point will underestimate the peak signal but give the same tumor growth ratio as the peak signal. The signal at 5 min was much lower than the peak signal, and the normalized signal at 5 min was much lower than 100%, implying marked underestimation of the peak signal. More importantly, the normalized signal at 5 min varied with days. It was especially low early after cell inoculation and increased with days consistent with the shortening of the peak time. As a result of the predominant underestimation on day 3, the tumor growth was greatly overestimated using the signal at 5 min as an indicator of tumor burden compared to using the peak signal. It is noteworthy that the signal at a fixed time point after d-luciferin injection relative to the peak signal may change with days after tumor cell inoculation and that quantitative assessments using different imaging timings may provide different estimates of tumor growth. In our tumor model, the peak time was about 10 min, except during the early period after cell inoculation. When the single-point imaging strategy is adopted, the signal at 10 min is likely to be used as an indicator of tumor burden. The normalized signal at 10 min was close to 100% on day 7 or later, but definitely lower before day 7. Consequently, the tumor growth was overestimated using the signal at 10 min compared to using the peak signal. The normalized signal at 15 min was definitely lower than 100%, but did not vary markedly over days, and thus the discrepancy in the growth rate between the signal at 15 min and the peak signal was relatively small. The optimal imaging timing to obtain an alternative to the peak signal may not necessarily correspond to the peak time determined for a well-established tumor.

The signal at a fixed time point after d-luciferin injection was closely correlated with the peak signal, irrespective of the time point used for analysis, apparently justifying the single-point imaging strategy. However, the signal at 5 min tended to be lower than the peak signal at low signal levels, which was attributable to the predominant discrepancy early after cell inoculation. A possible systematic discrepancy between the signal at a fixed time point and the peak signal should be considered, despite the high correlation between them. A high correlation does not necessarily guarantee exchangeability.

Since the time course of bioluminescence after d-luciferin injection changes with days after tumor cell inoculation and since the changes affect the observations of the tumor growth, sequential imaging for each measurement appears to be desirable. If the single-point imaging strategy is adopted to improve the throughput of the measurements, the possible effect of the changes in peak time on quantitative analysis should be considered. We recommend evaluating the relationship between the time course of signals after d-luciferin injection and the number of days after cell inoculation in preliminary experiments using a small number of animals.

The signal early after d-luciferin injection appears to be greatly affected by tumor blood flow. The peak signal is obtained at a time point when the delivery of d-luciferin is attained sufficiently, and it presumably reflects the luciferase activity relatively faithfully. The ratio of the signal at an early time point to the peak signal may serve as an indicator of tumor blood flow. Dynamic contrast-enhanced magnetic resonance imaging (MRI) allows the assessment of tumor blood flow; however, the assessment requires an MRI system, which is much more expensive than a system for BLI. In addition, because of the limited sensitivity to contrast materials, evaluating blood supply to small tumors by using dynamic contrast-enhanced MRI is difficult. The excellent sensitivity of BLI may be especially useful for assessing blood flow in small tumors. The delayed peak and the low normalized signal at 5 min observed in this study appear to imply insufficient vascularization of the tumor early after cell inoculation. Such prematurity may affect not only the quantitative analysis of in vivo BLI, but also the phenotype and therapeutic response of the tumor. One may use tumors for experiments after the tumors and their vasculature are established, and the peak time and normalized signal at 5 min reach plateaus. The evaluation of blood supply using sequential BLI may aid in characterizing tumor models.

In summary, the time course of bioluminescence signals after d-luciferin injection varies over days after cell inoculation in mice bearing subcutaneous tumors, and such variation should be considered when determining the imaging protocol and the method of quantitative analysis. Although the signal at a predetermined, fixed time point after d-luciferin injection is correlated with the peak signal, the time point used for analysis affects the correlation and the estimates of tumor growth. The time course after d-luciferin injection may provide an indicator of tumor blood flow and contribute to the assessment of tumor model animals.
